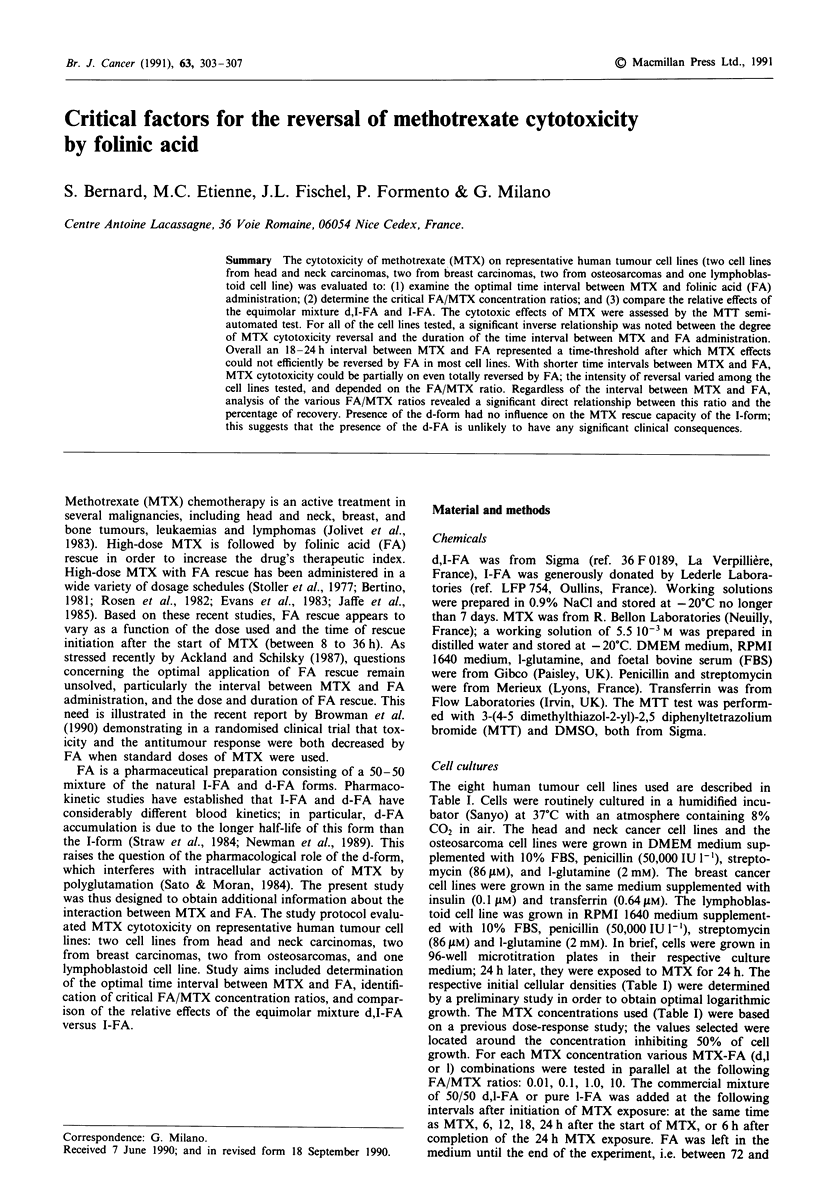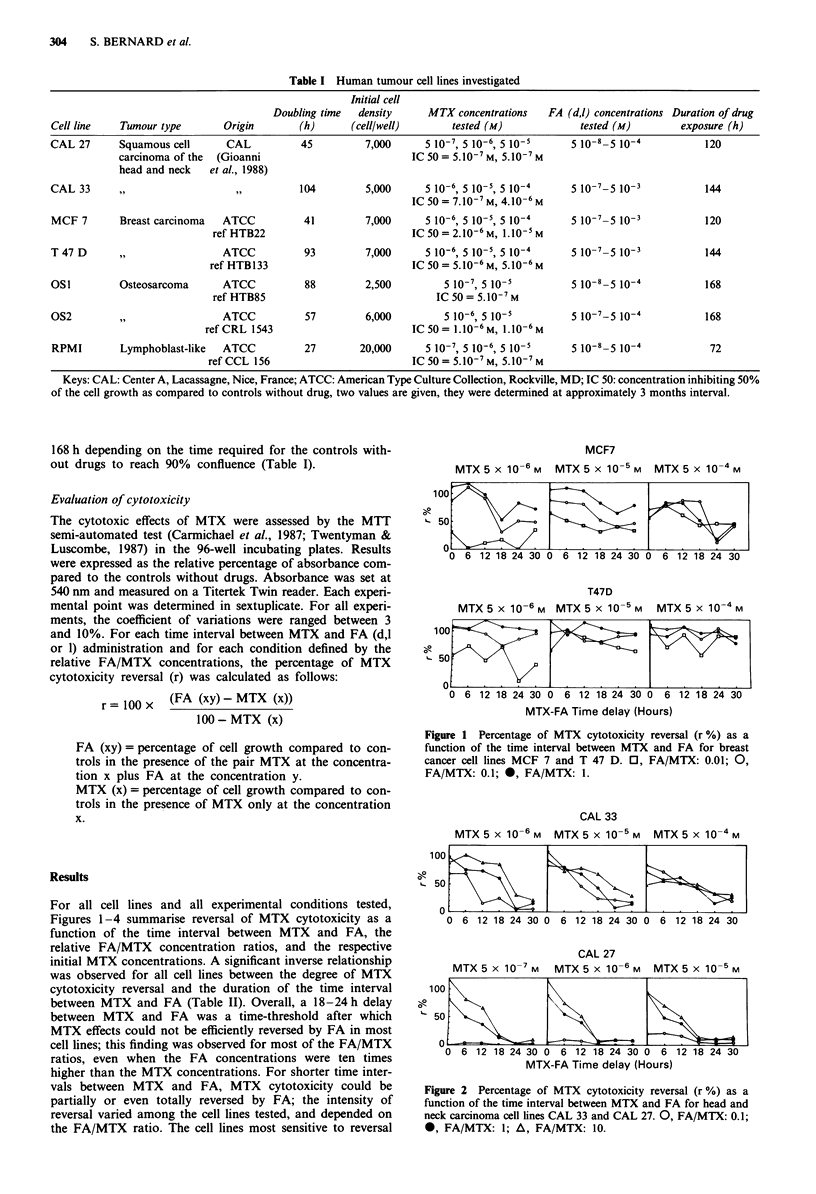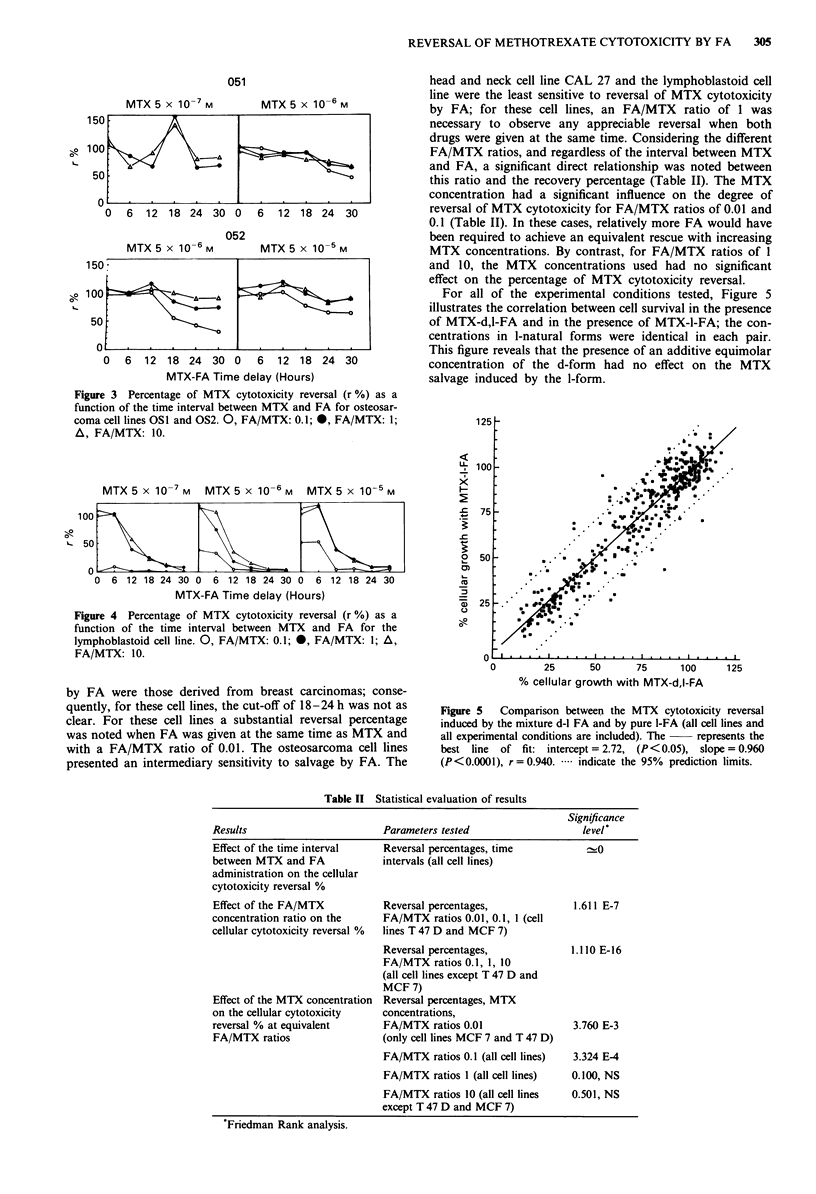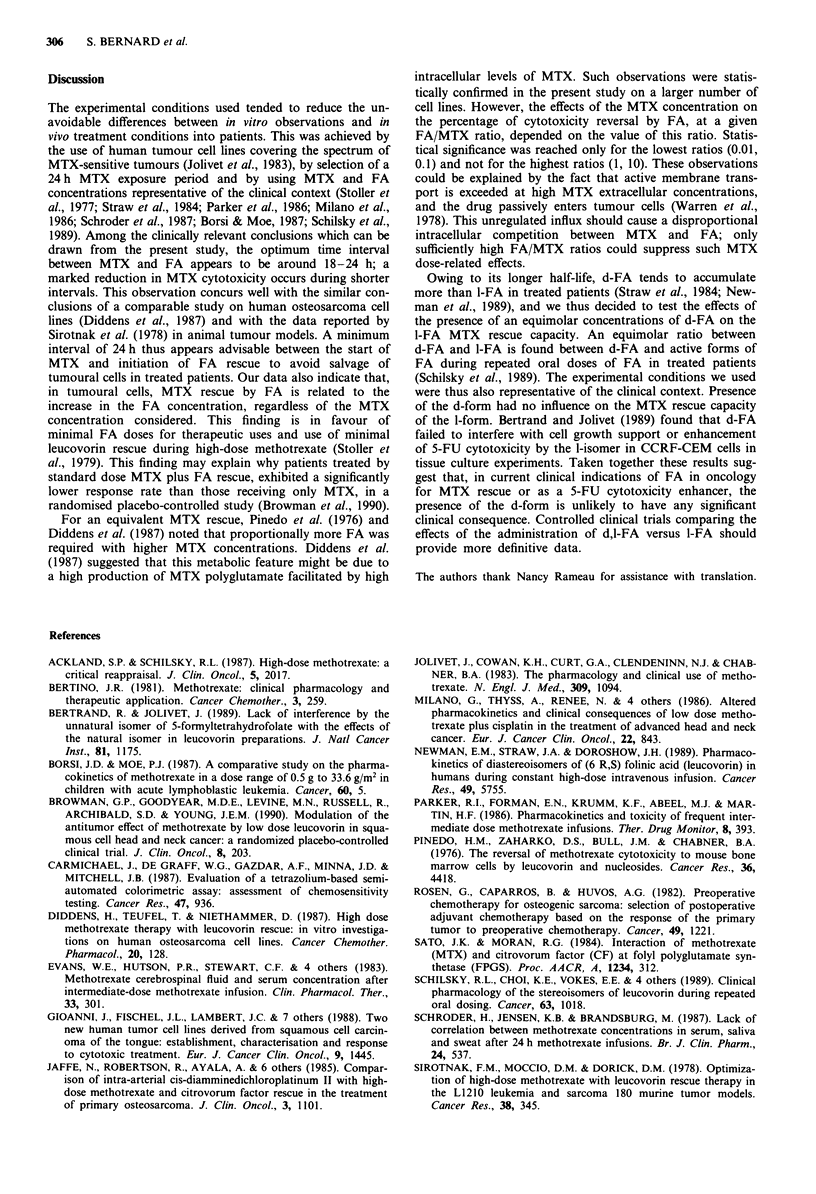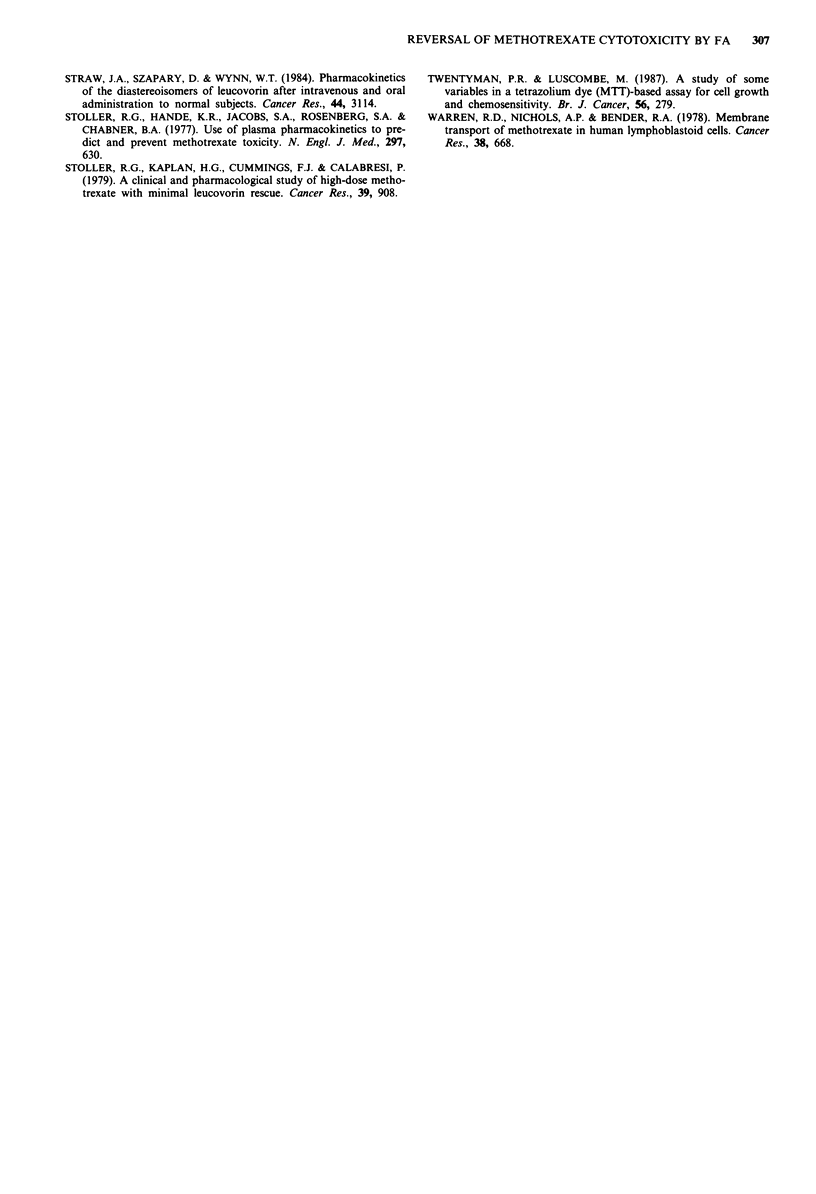# Critical factors for the reversal of methotrexate cytotoxicity by folinic acid.

**DOI:** 10.1038/bjc.1991.70

**Published:** 1991-02

**Authors:** S. Bernard, M. C. Etienne, J. L. Fischel, P. Formento, G. Milano

**Affiliations:** Centre Antoine Lacassagne, Nice, France.

## Abstract

The cytotoxicity of methotrexate (MTX) on representative human tumour cell lines (two cell lines from head and neck carcinomas, two from breast carcinomas, two from osteosarcomas and one lymphoblastoid cell line) was evaluated to: (1) examine the optimal time interval between MTX and folinic acid (FA) administration; (2) determine the critical FA/MTX concentration ratios; and (3) compare the relative effects of the equimolar mixture d,I-FA and I-FA. The cytotoxic effects of MTX were assessed by the MTT semi-automated test. For all of the cell lines tested, a significant inverse relationship was noted between the degree of MTX cytotoxicity reversal and the duration of the time interval between MTX and FA administration. Overall an 18-24 h interval between MTX and FA represented a time-threshold after which MTX effects could not efficiently be reversed by FA in most cell lines. With shorter time intervals between MTX and FA, MTX cytotoxicity could be partially on even totally reversed by FA; the intensity of reversal varied among the cell lines tested, and depended on the FA/MTX ratio. Regardless of the interval between MTX and FA, analysis of the various FA/MTX ratios revealed a significant direct relationship between this ratio and the percentage of recovery. Presence of the d-form had no influence on the MTX rescue capacity of the I-form; this suggests that the presence of the d-FA is unlikely to have any significant clinical consequences.


Br. J. Cancer (1991), 63, 303-307 © Macmillan Press Ltd., 1991

### Critical factors for the reversal of methotrexate cytotoxicity by folinic acid

S. Bernard, M.C. Etienne, J.L. Fischel, P. Formento & G. Milano

*Centre Antoine Lacassagne, 36 Voie Romaine, 06054 Nice Cedex, France.*

**Summary** The cytotoxicity of methotrexate (MTX) on representative human tumour cell lines (two cell lines from head and neck carcinomas, two from breast carcinomas, two from osteosarcomas and one lymphoblastoid cell line) was evaluated to: (1) examine the optimal time interval between MTX and folinic acid (FA) administration; (2) determine the critical FA/MTX concentration ratios; and (3) compare the relative effects of the equimolar mixture d,I-FA and I-FA. The cytotoxic effects of MTX were assessed by the MTT semi-automated test. For all of the cell lines tested, a significant inverse relationship was noted between the degree of MTX cytotoxicity reversal and the duration of the time interval between MTX and FA administration. Overall an 18-24 h interval between MTX and FA represented a time-threshold after which MTX effects could not efficiently be reversed by FA in most cell lines. With shorter time intervals between MTX and FA, MTX cytotoxicity could be partially on even totally reversed by FA; the intensity of reversal varied among the cell lines tested, and depended on the FA/MTX ratio. Regardless of the interval between MTX and FA, analysis of the various FA/MTX ratios revealed a significant direct relationship between this ratio and the percentage of recovery. Presence of the d-form had no influence on the MTX rescue capacity of the I-form; this suggests that the presence of the d-FA is unlikely to have any significant clinical consequences.

Methotrexate (MTX) chemotherapy is an active treatment in several malignancies, including head and neck, breast, and bone tumours, leukaemias and lymphomas (Jolivet *et al.*, 1983). High-dose MTX is followed by folinic acid (FA) rescue in order to increase the drug's therapeutic index. High-dose MTX with FA rescue has been administered in a wide variety of dosage schedules (Stoller *et al.*, 1977; Bertino, 1981; Rosen *et al.*, 1982; Evans *et al.*, 1983; Jaffe *et al.*, 1985). Based on these recent studies, FA rescue appears to vary as a function of the dose used and the time of rescue initiation after the start of MTX (between 8 to 36 h). As stressed recently by Ackland and Schilsky (1987), questions concerning the optimal application of FA rescue remain unsolved, particularly the interval between MTX and FA administration, and the dose and duration of FA rescue. This need is illustrated in the recent report by Browman *et al.* (1990) demonstrating in a randomised clinical trial that toxicity and the antitumour response were both decreased by FA when standard doses of MTX were used.

FA is a pharmaceutical preparation consisting of a 50-50 mixture of the natural I-FA and d-FA forms. Pharmacokinetic studies have established that I-FA and d-FA have considerably different blood kinetics; in particular, d-FA accumulation is due to the longer half-life of this form than the I-form (Straw *et al.*, 1984; Newman *et al.*, 1989). This raises the question of the pharmacological role of the d-form, which interferes with intracellular activation of MTX by polyglutamation (Sato & Moran, 1984). The present study was thus designed to obtain additional information about the interaction between MTX and FA. The study protocol evaluated MTX cytotoxicity on representative human tumour cell lines: two cell lines from head and neck carcinomas, two from breast carcinomas, two from osteosarcomas, and one lymphoblastoid cell line. Study aims included determination of the optimal time interval between MTX and FA, identification of critical FA/MTX concentration ratios, and comparison of the relative effects of the equimolar mixture d,I-FA versus I-FA.

Correspondence: G. Milano.
Received 7 June 1990; and in revised form 18 September 1990.

### Material and methods

### *Chemicals*

d,I-FA was from Sigma (ref. 36 F 0189, La Verpillière, France), I-FA was generously donated by Lederle Laboratories (ref. LFP 754, Oullins, France). Working solutions were prepared in 0.9% NaCl and stored at $-20$°C no longer than 7 days. MTX was from R. Bellon Laboratories (Neuilly, France); a working solution of $5.5\ 10^{-3}$ M was prepared in distilled water and stored at $-20$°C. DMEM medium, RPMI 1640 medium, l-glutamine, and foetal bovine serum (FBS) were from Gibco (Paisley, UK). Penicillin and streptomycin were from Merieux (Lyons, France). Transferrin was from Flow Laboratories (Irvin, UK). The MTT test was performed with 3-(4-5 dimethylthiazol-2-yl)-2,5 diphenyltetrazolium bromide (MTT) and DMSO, both from Sigma.

### *Cell cultures*

The eight human tumour cell lines used are described in Table I. Cells were routinely cultured in a humidified incubator (Sanyo) at 37°C with an atmosphere containing 8% $CO_2$ in air. The head and neck cancer cell lines and the osteosarcoma cell lines were grown in DMEM medium supplemented with 10% FBS, penicillin (50,000 IU $l^{-1}$), streptomycin (86 μM), and l-glutamine (2 mM). The breast cancer cell lines were grown in the same medium supplemented with insulin (0.1 μM) and transferrin (0.64 μM). The lymphoblastoid cell line was grown in RPMI 1640 medium supplemented with 10% FBS, penicillin (50,000 IU $l^{-1}$), streptomycin (86 μM) and l-glutamine (2 mM). In brief, cells were grown in 96-well microtitration plates in their respective culture medium; 24 h later, they were exposed to MTX for 24 h. The respective initial cellular densities (Table I) were determined by a preliminary study in order to obtain optimal logarithmic growth. The MTX concentrations used (Table I) were based on a previous dose-response study; the values selected were located around the concentration inhibiting 50% of cell growth. For each MTX concentration various MTX-FA (d,l or l) combinations were tested in parallel at the following FA/MTX ratios: 0.01, 0.1, 1.0, 10. The commercial mixture of 50/50 d,l-FA or pure l-FA was added at the following intervals after initiation of MTX exposure: at the same time as MTX, 6, 12, 18, 24 h after the start of MTX, or 6 h after completion of the 24 h MTX exposure. FA was left in the medium until the end of the experiment, i.e. between 72 and

**Table I** Human tumour cell lines investigated

| Cell line | Tumour type | Origin | Doubling time (h) | Initial cell density (cell/well) | MTX concentrations tested (M) | FA (d,l) concentrations tested (M) | Duration of drug exposure (h) |
|---|---|---|---|---|---|---|---|
| CAL 27 | Squamous cell carcinoma of the head and neck | CAL (Gioanni et al., 1988) | 45 | 7,000 | $5\ 10^{-7}$, $5\ 10^{-6}$, $5\ 10^{-5}$ IC 50 = $5.10^{-7}$ M, $5.10^{-7}$ M | $5\ 10^{-8}$–$5\ 10^{-4}$ | 120 |
| CAL 33 | „ | „ | 104 | 5,000 | $5\ 10^{-6}$, $5\ 10^{-5}$, $5\ 10^{-4}$ IC 50 = $7.10^{-7}$ M, $4.10^{-6}$ M | $5\ 10^{-7}$–$5\ 10^{-3}$ | 144 |
| MCF 7 | Breast carcinoma | ATCC ref HTB22 | 41 | 7,000 | $5\ 10^{-6}$, $5\ 10^{-5}$, $5\ 10^{-4}$ IC 50 = $2.10^{-6}$ M, $1.10^{-5}$ M | $5\ 10^{-7}$–$5\ 10^{-3}$ | 120 |
| T 47 D | „ | ATCC ref HTB133 | 93 | 7,000 | $5\ 10^{-6}$, $5\ 10^{-5}$, $5\ 10^{-4}$ IC 50 = $5.10^{-6}$ M, $5.10^{-6}$ M | $5\ 10^{-7}$–$5\ 10^{-3}$ | 144 |
| OS1 | Osteosarcoma | ATCC ref HTB85 | 88 | 2,500 | $5\ 10^{-7}$, $5\ 10^{-5}$ IC 50 = $5.10^{-7}$ M | $5\ 10^{-8}$–$5\ 10^{-4}$ | 168 |
| OS2 | „ | ATCC ref CRL 1543 | 57 | 6,000 | $5\ 10^{-6}$, $5\ 10^{-5}$ IC 50 = $1.10^{-6}$ M, $1.10^{-6}$ M | $5\ 10^{-7}$–$5\ 10^{-4}$ | 168 |
| RPMI | Lymphoblast-like | ATCC ref CCL 156 | 27 | 20,000 | $5\ 10^{-7}$, $5\ 10^{-6}$, $5\ 10^{-5}$ IC 50 = $5.10^{-7}$ M, $5.10^{-7}$ M | $5\ 10^{-8}$–$5\ 10^{-4}$ | 72 |

Keys: CAL: Center A, Lacassagne, Nice, France; ATCC: American Type Culture Collection, Rockville, MD; IC 50: concentration inhibiting 50% of the cell growth as compared to controls without drug, two values are given, they were determined at approximately 3 months interval.

168 h depending on the time required for the controls without drugs to reach 90% confluence (Table I).

### Evaluation of cytotoxicity

The cytotoxic effects of MTX were assessed by the MTT semi-automated test (Carmichael et al., 1987; Twentyman & Luscombe, 1987) in the 96-well incubating plates. Results were expressed as the relative percentage of absorbance compared to the controls without drugs. Absorbance was set at 540 nm and measured on a Titertek Twin reader. Each experimental point was determined in sextuplicate. For all experiments, the coefficient of variations were ranged between 3 and 10%. For each time interval between MTX and FA (d,l or l) administration and for each condition defined by the relative FA/MTX concentrations, the percentage of MTX cytotoxicity reversal (r) was calculated as follows:

$$r = 100 \times \frac{(FA\ (xy) - MTX\ (x))}{100 - MTX\ (x)}$$

FA (xy) = percentage of cell growth compared to controls in the presence of the pair MTX at the concentration x plus FA at the concentration y.
MTX (x) = percentage of cell growth compared to controls in the presence of MTX only at the concentration x.

### Results

For all cell lines and all experimental conditions tested, Figures 1–4 summarise reversal of MTX cytotoxicity as a function of the time interval between MTX and FA, the relative FA/MTX concentration ratios, and the respective initial MTX concentrations. A significant inverse relationship was observed for all cell lines between the degree of MTX cytotoxicity reversal and the duration of the time interval between MTX and FA (Table II). Overall, a 18–24 h delay between MTX and FA was a time-threshold after which MTX effects could not be efficiently reversed by FA in most cell lines; this finding was observed for most of the FA/MTX ratios, even when the FA concentrations were ten times higher than the MTX concentrations. For shorter time intervals between MTX and FA, MTX cytotoxicity could be partially or even totally reversed by FA; the intensity of reversal varied among the cell lines tested, and depended on the FA/MTX ratio. The cell lines most sensitive to reversal

**Figure 1** Percentage of MTX cytotoxicity reversal (r %) as a function of the time interval between MTX and FA for breast cancer cell lines MCF 7 and T 47 D. □, FA/MTX: 0.01; ○, FA/MTX: 0.1; ●, FA/MTX: 1.

**Figure 2** Percentage of MTX cytotoxicity reversal (r %) as a function of the time interval between MTX and FA for head and neck carcinoma cell lines CAL 33 and CAL 27. ○, FA/MTX: 0.1; ●, FA/MTX: 1; △, FA/MTX: 10.

**Figure 3** Percentage of MTX cytotoxicity reversal (r %) as a function of the time interval between MTX and FA for osteosarcoma cell lines OS1 and OS2. O, FA/MTX: 0.1; ●, FA/MTX: 1; Δ, FA/MTX: 10.

**Figure 4** Percentage of MTX cytotoxicity reversal (r %) as a function of the time interval between MTX and FA for the lymphoblastoid cell line. O, FA/MTX: 0.1; ●, FA/MTX: 1; Δ, FA/MTX: 10.

by FA were those derived from breast carcinomas; consequently, for these cell lines, the cut-off of 18–24 h was not as clear. For these cell lines a substantial reversal percentage was noted when FA was given at the same time as MTX and with a FA/MTX ratio of 0.01. The osteosarcoma cell lines presented an intermediary sensitivity to salvage by FA. The head and neck cell line CAL 27 and the lymphoblastoid cell line were the least sensitive to reversal of MTX cytotoxicity by FA; for these cell lines, an FA/MTX ratio of 1 was necessary to observe any appreciable reversal when both drugs were given at the same time. Considering the different FA/MTX ratios, and regardless of the interval between MTX and FA, a significant direct relationship was noted between this ratio and the recovery percentage (Table II). The MTX concentration had a significant influence on the degree of reversal of MTX cytotoxicity for FA/MTX ratios of 0.01 and 0.1 (Table II). In these cases, relatively more FA would have been required to achieve an equivalent rescue with increasing MTX concentrations. By contrast, for FA/MTX ratios of 1 and 10, the MTX concentrations used had no significant effect on the percentage of MTX cytotoxicity reversal.

For all of the experimental conditions tested, Figure 5 illustrates the correlation between cell survival in the presence of MTX-d,l-FA and in the presence of MTX-l-FA; the concentrations in l-natural forms were identical in each pair. This figure reveals that the presence of an additive equimolar concentration of the d-form had no effect on the MTX salvage induced by the l-form.

**Figure 5** Comparison between the MTX cytotoxicity reversal induced by the mixture d-l FA and by pure l-FA (all cell lines and all experimental conditions are included). The —— represents the best line of fit: intercept = 2.72, ($P < 0.05$), slope = 0.960 ($P < 0.0001$), $r = 0.940$. ···· indicate the 95% prediction limits.

**Table II** Statistical evaluation of results

| *Results* | *Parameters tested* | *Significance level** |
|---|---|---|
| Effect of the time interval between MTX and FA administration on the cellular cytotoxicity reversal % | Reversal percentages, time intervals (all cell lines) | ≃0 |
| Effect of the FA/MTX concentration ratio on the cellular cytotoxicity reversal % | Reversal percentages, FA/MTX ratios 0.01, 0.1, 1 (cell lines T 47 D and MCF 7) | 1.611 E-7 |
| | Reversal percentages, FA/MTX ratios 0.1, 1, 10 (all cell lines except T 47 D and MCF 7) | 1.110 E-16 |
| Effect of the MTX concentration on the cellular cytotoxicity reversal % at equivalent FA/MTX ratios | Reversal percentages, MTX concentrations, FA/MTX ratios 0.01 (only cell lines MCF 7 and T 47 D) | 3.760 E-3 |
| | FA/MTX ratios 0.1 (all cell lines) | 3.324 E-4 |
| | FA/MTX ratios 1 (all cell lines) | 0.100, NS |
| | FA/MTX ratios 10 (all cell lines except T 47 D and MCF 7) | 0.501, NS |

*Friedman Rank analysis.

### Discussion

The experimental conditions used tended to reduce the unavoidable differences between *in vitro* observations and *in vivo* treatment conditions into patients. This was achieved by the use of human tumour cell lines covering the spectrum of MTX-sensitive tumours (Jolivet *et al.*, 1983), by selection of a 24 h MTX exposure period and by using MTX and FA concentrations representative of the clinical context (Stoller *et al.*, 1977; Straw *et al.*, 1984; Parker *et al.*, 1986; Milano *et al.*, 1986; Schroder *et al.*, 1987; Borsi & Moe, 1987; Schilsky *et al.*, 1989). Among the clinically relevant conclusions which can be drawn from the present study, the optimum time interval between MTX and FA appears to be around 18–24 h; a marked reduction in MTX cytotoxicity occurs during shorter intervals. This observation concurs well with the similar conclusions of a comparable study on human osteosarcoma cell lines (Diddens *et al.*, 1987) and with the data reported by Sirotnak *et al.* (1978) in animal tumour models. A minimum interval of 24 h thus appears advisable between the start of MTX and initiation of FA rescue to avoid salvage of tumoural cells in treated patients. Our data also indicate that, in tumoural cells, MTX rescue by FA is related to the increase in the FA concentration, regardless of the MTX concentration considered. This finding is in favour of minimal FA doses for therapeutic uses and use of minimal leucovorin rescue during high-dose methotrexate (Stoller *et al.*, 1979). This finding may explain why patients treated by standard dose MTX plus FA rescue, exhibited a significantly lower response rate than those receiving only MTX, in a randomised placebo-controlled study (Browman *et al.*, 1990).

For an equivalent MTX rescue, Pinedo *et al.* (1976) and Diddens *et al.* (1987) noted that proportionally more FA was required with higher MTX concentrations. Diddens *et al.* (1987) suggested that this metabolic feature might be due to a high production of MTX polyglutamate facilitated by high intracellular levels of MTX. Such observations were statistically confirmed in the present study on a larger number of cell lines. However, the effects of the MTX concentration on the percentage of cytotoxicity reversal by FA, at a given FA/MTX ratio, depended on the value of this ratio. Statistical significance was reached only for the lowest ratios (0.01, 0.1) and not for the highest ratios (1, 10). These observations could be explained by the fact that active membrane transport is exceeded at high MTX extracellular concentrations, and the drug passively enters tumour cells (Warren *et al.*, 1978). This unregulated influx should cause a disproportional intracellular competition between MTX and FA; only sufficiently high FA/MTX ratios could suppress such MTX dose-related effects.

Owing to its longer half-life, d-FA tends to accumulate more than l-FA in treated patients (Straw *et al.*, 1984; Newman *et al.*, 1989), and we thus decided to test the effects of the presence of an equimolar concentrations of d-FA on the l-FA MTX rescue capacity. An equimolar ratio between d-FA and l-FA is found between d-FA and active forms of FA during repeated oral doses of FA in treated patients (Schilsky *et al.*, 1989). The experimental conditions we used were thus also representative of the clinical context. Presence of the d-form had no influence on the MTX rescue capacity of the l-form. Bertrand and Jolivet (1989) found that d-FA failed to interfere with cell growth support or enhancement of 5-FU cytotoxicity by the l-isomer in CCRF-CEM cells in tissue culture experiments. Taken together these results suggest that, in current clinical indications of FA in oncology for MTX rescue or as a 5-FU cytotoxicity enhancer, the presence of the d-form is unlikely to have any significant clinical consequence. Controlled clinical trials comparing the effects of the administration of d,l-FA versus l-FA should provide more definitive data.

The authors thank Nancy Rameau for assistance with translation.

### References

ACKLAND, S.P. & SCHILSKY, R.L. (1987). High-dose methotrexate: a critical reappraisal. *J. Clin. Oncol.*, **5**, 2017.

BERTINO, J.R. (1981). Methotrexate: clinical pharmacology and therapeutic application. *Cancer Chemother.*, **3**, 259.

BERTRAND, R. & JOLIVET, J. (1989). Lack of interference by the unnatural isomer of 5-formyltetrahydrofolate with the effects of the natural isomer in leucovorin preparations. *J. Natl Cancer Inst.*, **81**, 1175.

BORSI, J.D. & MOE, P.J. (1987). A comparative study on the pharmacokinetics of methotrexate in a dose range of 0.5 g to 33.6 g/m$^2$ in children with acute lymphoblastic leukemia. *Cancer*, **60**, 5.

BROWMAN, G.P., GOODYEAR, M.D.E., LEVINE, M.N., RUSSELL, R., ARCHIBALD, S.D. & YOUNG, J.E.M. (1990). Modulation of the antitumor effect of methotrexate by low dose leucovorin in squamous cell head and neck cancer: a randomized placebo-controlled clinical trial. *J. Clin. Oncol.*, **8**, 203.

CARMICHAEL, J., DE GRAFF, W.G., GAZDAR, A.F., MINNA, J.D. & MITCHELL, J.B. (1987). Evaluation of a tetrazolium-based semiautomated colorimetric assay: assessment of chemosensitivity testing. *Cancer Res.*, **47**, 936.

DIDDENS, H., TEUFEL, T. & NIETHAMMER, D. (1987). High dose methotrexate therapy with leucovorin rescue: in vitro investigations on human osteosarcoma cell lines. *Cancer Chemother. Pharmacol.*, **20**, 128.

EVANS, W.E., HUTSON, P.R., STEWART, C.F. & 4 others (1983). Methotrexate cerebrospinal fluid and serum concentration after intermediate-dose methotrexate infusion. *Clin. Pharmacol. Ther.*, **33**, 301.

GIOANNI, J., FISCHEL, J.L., LAMBERT, J.C. & 7 others (1988). Two new human tumor cell lines derived from squamous cell carcinoma of the tongue: establishment, characterisation and response to cytotoxic treatment. *Eur. J. Cancer Clin. Oncol.*, **9**, 1445.

JAFFE, N., ROBERTSON, R., AYALA, A. & 6 others (1985). Comparison of intra-arterial cis-diamminedichloroplatinum II with high-dose methotrexate and citrovorum factor rescue in the treatment of primary osteosarcoma. *J. Clin. Oncol.*, **3**, 1101.

JOLIVET, J., COWAN, K.H., CURT, G.A., CLENDENINN, N.J. & CHABNER, B.A. (1983). The pharmacology and clinical use of methotrexate. *N. Engl. J. Med.*, **309**, 1094.

MILANO, G., THYSS, A., RENEE, N. & 4 others (1986). Altered pharmacokinetics and clinical consequences of low dose methotrexate plus cisplatin in the treatment of advanced head and neck cancer. *Eur. J. Cancer Clin. Oncol.*, **22**, 843.

NEWMAN, E.M., STRAW, J.A. & DOROSHOW, J.H. (1989). Pharmacokinetics of diastereoisomers of (6 R,S) folinic acid (leucovorin) in humans during constant high-dose intravenous infusion. *Cancer Res.*, **49**, 5755.

PARKER, R.I., FORMAN, E.N., KRUMM, K.F., ABEEL, M.J. & MARTIN, H.F. (1986). Pharmacokinetics and toxicity of frequent intermediate dose methotrexate infusions. *Ther. Drug Monitor*, **8**, 393.

PINEDO, H.M., ZAHARKO, D.S., BULL, J.M. & CHABNER, B.A. (1976). The reversal of methotrexate cytotoxicity to mouse bone marrow cells by leucovorin and nucleosides. *Cancer Res.*, **36**, 4418.

ROSEN, G., CAPARROS, B. & HUVOS, A.G. (1982). Preoperative chemotherapy for osteogenic sarcoma: selection of postoperative adjuvant chemotherapy based on the response of the primary tumor to preoperative chemotherapy. *Cancer*, **49**, 1221.

SATO, J.K. & MORAN, R.G. (1984). Interaction of methotrexate (MTX) and citrovorum factor (CF) at folyl polyglutamate synthetase (FPGS). *Proc. AACR, A*, **1234**, 312.

SCHILSKY, R.L., CHOI, K.E., VOKES, E.E. & 4 others (1989). Clinical pharmacology of the stereoisomers of leucovorin during repeated oral dosing. *Cancer*, **63**, 1018.

SCHRODER, H., JENSEN, K.B. & BRANDSBURG, M. (1987). Lack of correlation between methotrexate concentrations in serum, saliva and sweat after 24 h methotrexate infusions. *Br. J. Clin. Pharm.*, **24**, 537.

SIROTNAK, F.M., MOCCIO, D.M. & DORICK, D.M. (1978). Optimization of high-dose methotrexate with leucovorin rescue therapy in the L1210 leukemia and sarcoma 180 murine tumor models. *Cancer Res.*, **38**, 345.

STRAW, J.A., SZAPARY, D. & WYNN, W.T. (1984). Pharmacokinetics of the diastereoisomers of leucovorin after intravenous and oral administration to normal subjects. *Cancer Res.*, **44**, 3114.

STOLLER, R.G., HANDE, K.R., JACOBS, S.A., ROSENBERG, S.A. & CHABNER, B.A. (1977). Use of plasma pharmacokinetics to predict and prevent methotrexate toxicity. *N. Engl. J. Med.*, **297**, 630.

STOLLER, R.G., KAPLAN, H.G., CUMMINGS, F.J. & CALABRESI, P. (1979). A clinical and pharmacological study of high-dose methotrexate with minimal leucovorin rescue. *Cancer Res.*, **39**, 908.

TWENTYMAN, P.R. & LUSCOMBE, M. (1987). A study of some variables in a tetrazolium dye (MTT)-based assay for cell growth and chemosensitivity. *Br. J. Cancer*, **56**, 279.

WARREN, R.D., NICHOLS, A.P. & BENDER, R.A. (1978). Membrane transport of methotrexate in human lymphoblastoid cells. *Cancer Res.*, **38**, 668.